# Immunoglobulin M Nephropathy: A Diagnostic Dilemma Between Minimal Change Disease and Focal Segmental Glomerulosclerosis

**DOI:** 10.7759/cureus.84148

**Published:** 2025-05-15

**Authors:** Gautam Agrawal, Bhawna Agarwal, Kunal Sonavane, Pallavi Shirsat

**Affiliations:** 1 Nephrology, Independence Health System, Greensburg, USA; 2 Internal Medicine, University of Pittsburgh Medical Center, McKeesport Hospital, McKeesport, USA; 3 Internal Medicine, Willis-Knighton Medical Center, Bossier City, USA; 4 Nephrology, Minden Medical Center, Minden, USA

**Keywords:** chronic kidney diseases, focal segmental glomerulosclerosis, hypertension, igm nephropathy, minimal change disease, secondary hypertension, subnephrotic proteinuria

## Abstract

Immunoglobulin M nephropathy (IgMN) is an idiopathic glomerulonephritis characterized by diffuse IgM deposits in the mesangium and mesangial hypercellularity. It has been a controversial diagnosis since it was initially described, as IgMN shares features with both minimal change disease (MCD) and focal segmental glomerulosclerosis (FSGS). However, studies have shown that IgMN is a distinct clinicopathological diagnosis comprising patients with predominant mesangial IgM deposits and who do not meet established criteria for MCD and FSGS. We present a case of a 24-year-old male presenting with hypertension and proteinuria, with kidney biopsy showing concern for IgMN. These patients carry a significant risk of developing nephrotic syndrome, renal insufficiency, and progression to end-stage renal failure. This underscores the importance of timely diagnosis of IgMN and therapeutic intervention to prevent kidney function decline. Management options include corticosteroids or calcineurin inhibitors, with some evidence on the use of rituximab for refractory cases. There is a need for further research on IgMN to establish standardized treatment protocols and long-term outcomes for patients with IgMN.

## Introduction

Immunoglobulin M nephropathy (IgMN) is an idiopathic glomerulonephritis characterized by mesangial hypercellularity and diffuse mesangial deposition of IgM [1]. The reported frequency of IgMN has been variable, ranging from 1.8% to 18.5% in native kidney biopsies [2]. It has remained a controversial diagnosis since it was first described in the 1970s, as IgMN shares features with both minimal change disease (MCD) and focal segmental glomerulosclerosis (FSGS) [2]. IgMN is considered to fall in the spectrum between MCD and FSGS, but it is evident that there is a distinct group of patients, identifiable by immunohistochemistry, who exhibit predominant mesangial IgM deposition and do not meet the established diagnostic criteria for either MCD or FSGS [2]. Clinically, IgMN can present with nephrotic syndrome, but it also shows more mesangial proliferation and matrix expansion, which are not typical of MCD. Histologically, IgMN exhibits more tubular atrophy and interstitial fibrosis compared to MCD, aligning it closer to FSGS [3].

The Mayo Clinic/Renal Pathology Society provides specific guidelines for diagnosing IgMN. Typically, IgMN presents mesangial hypercellularity and diffuse mesangial IgM deposits, but IgMN can be associated with tubular atrophy and interstitial fibrosis, which are significant predictors of renal insufficiency and considered to be poor prognostic markers [4].

We present a case of a 24-year-old male who presented with hypertension, found to have proteinuria, and further evaluation with kidney biopsy showed concern for IgMN.

## Case presentation

A 24-year-old healthy male was referred to the nephrology clinic after being found to have hypertension. He denied any significant past medical history, including chronic kidney disease. He was born at full term. His father had a notable history of hypertension and congestive heart failure and died at the age of 50 from an unknown cause. The patient denied any known family history of chronic kidney disease.

On initial examination, the patient weighed 232 lbs. His blood pressure was 130/84 mmHg in the left upper arm. He appeared well-built, alert, and oriented. Pulmonary examination revealed clear breath sounds bilaterally, with no crackles or wheezes. Cardiac examination revealed regular heart sounds. There was no peripheral edema, rash, or tattoos noted.

Initial laboratory workup revealed a serum creatinine of 1.3 mg/dL, with an estimated glomerular filtration rate (eGFR) of 68 mL/min/1.73 m². Urinalysis showed trace proteinuria and microscopic hematuria. A 24-hour urine collection revealed proteinuria of 248 mg/day. Serologic testing was negative for antinuclear antibodies and double-stranded DNA antibodies, with normal complement levels. Other laboratory results were unremarkable (Table 1).

**Table 1:** Laboratory values. ANA: antinuclear antibody; Anti-dsDNA: anti-double-stranded DNA.

Laboratory tests	Results	Reference range
24-hour protein	248	<150 mg
Serum albumin	4.6	3.5-5.7 gm/dl
Blood urea nitrogen (BUN)	17	7-25 mg/dl
Creatinine	1.3	0.6-1.2 mg/dl
Calcium	9.8	8.6-10.3 mg/dl
Hemoglobin	15.6	11.7-15.8 gm/dl
ANA titer	Negative	Negative
Anti-dsDNA	Negative	<25 - negative
Hemoglobin A1c	5.5	5.7-6.4 (prediabetic) %
Hepatitis B surface antigen	Non-reactive	Non-reactive
Hepatitis C antibody	Non-reactive	Non-reactive

**Figure 1:**
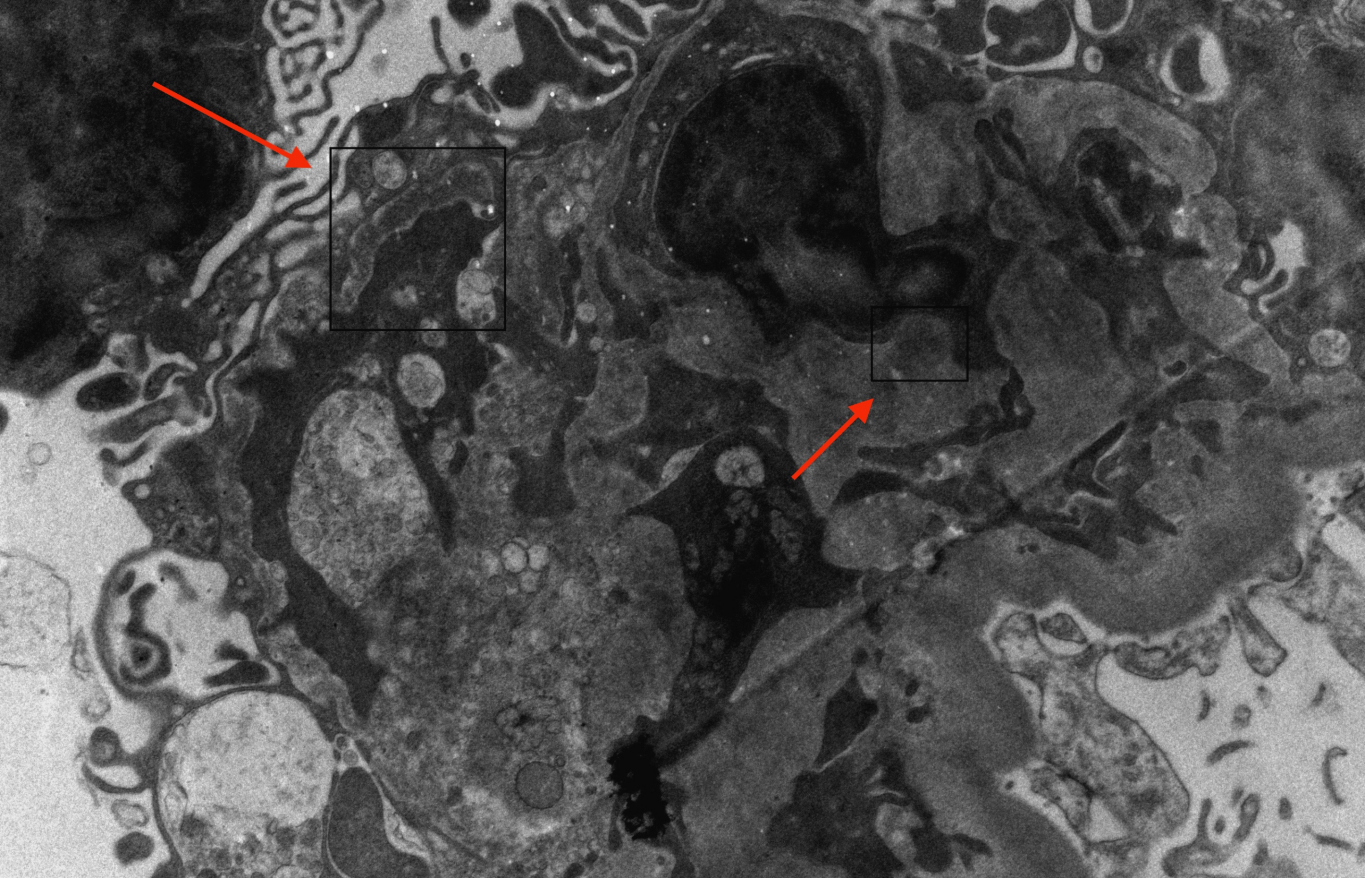
Kidney biopsy image. Red arrows showing mesangial deposits.

The patient underwent a kidney biopsy, which revealed minimal histopathological changes with mildly increased mesangial cellularity and weak mesangial IgM, C3, and kappa-dominant deposits. Electron microscopy showed 15% podocyte foot process effacement and equivocal mesangial electron-dense deposits (Figure 1), with focal endothelial hypertrophy and questionable basement membrane duplication. No chronic changes, glomerulosclerosis, or evidence of arterionephrosclerosis, interstitial fibrosis, or tubular atrophy were observed. These findings were concerning for IgM nephropathy.

He was initiated on aggressive blood pressure control with lisinopril, later supplemented with amlodipine, resulting in improvement in both blood pressure and proteinuria. His proteinuria has improved to 154 mg in the most recent 24-hour urine collection. The current management plan involves continued monitoring of proteinuria, with consideration of corticosteroid therapy in the event of future flares.

## Discussion

Our patient presented with hypertension at a young age, with further evaluation showing proteinuria, which necessitated further evaluation with kidney biopsy, and the diagnosis of IgMN was established. We would like to highlight the importance of appropriate diagnostic evaluation for patients with hypertension at a young age and proteinuria with renal biopsy, given that timely therapeutic intervention in these patients is of utmost importance to prevent kidney function decline.

IgM nephropathy can present as sub-nephrotic range proteinuria, hematuria, or nephrotic syndrome, which is commonly characterized by steroid dependence or resistance, with a significant risk of progression to end-stage renal disease. IgMN shares clinical and histopathological features with MCD and FSGS [4]. Key differences between IgM nephropathy, MCD, and FSGS are outlined in Table 2.

**Table 2:** Differential features between IgM nephropathy, MCD, and FSGS.

Category	IgM nephropathy	Minimal change disease (MCD)	Focal segmental glomerulosclerosis (FSGS)
Clinical presentation	Nephrotic syndrome, non-nephrotic range proteinuria, or hematuria	Nephrotic syndrome	Nephrotic syndrome with hypertension and renal insufficiency
Biopsy findings	Diffuse IgM deposits in mesangium and mesangial proliferation	Normal glomeruli on light microscopy, effacement of podocyte foot processes on electron microscopy	Sclerosis affecting a portion of the glomerulus, effacement of the podocyte foot process
Response to treatment	Variable response to steroids, higher rate of steroid dependence and resistance as compared to MCD	High response rate to steroids	Poor response rate to steroids, often requires other immunosuppressants

In a study, when IgMN was divided into subtypes, patients with FSGS-like IgMN had lower eGFR and higher proteinuria compared to those with MCD-like IgMN. Diagnosis of hypertension at the time of kidney biopsy was identified as a predictor of a ≥20% decline in eGFR over two years in IgMN patients [5]. Investigators suggest that repeat biopsies in patients with IgMN often show FSGS despite ongoing mesangial IgM deposition [2]. Researchers have shown that over 15 years, 36% of patients with IgMN developed renal insufficiency, and 23% progressed to end-stage renal failure (ESRD). Interstitial fibrosis was found to have the strongest prognostic value among histological parameters [1].

Renin-angiotensin system inhibitors are recommended as part of the standard treatment for IgMN. Treatment options also include corticosteroids or calcineurin inhibitors for nephrotic range proteinuria, but steroid dependence and resistance are higher for IgMN as compared to MCD. Research has shown that among IgMN patients with nephrotic syndrome, 29% were steroid-resistant, while 80% of those who initially responded to steroids became steroid-dependent [1]. Rituximab has also demonstrated efficacy in inducing remission in refractory cases of IgMN, making it a valuable therapeutic option. Rituximab has shown efficacy in treating steroid-dependent or frequently relapsing nephrotic syndrome in MCD and FSGS, reducing relapse rates and steroid dependence. This aligns with the successful use of rituximab in the IgMN case [6,7]. Further research and clinical trials are needed to establish its role more definitively in the management of IgMN.

## Conclusions

This case underscores the critical importance of thorough diagnostic evaluation in young patients presenting with hypertension and proteinuria. Early consideration of renal biopsy can lead to timely diagnosis of underlying glomerular diseases such as IgM nephropathy. Prompt identification and appropriate management are essential to prevent progression of renal dysfunction and to optimize long-term outcomes. There is a need for robust clinical trials on IgMN to establish standardized treatment protocols and long-term outcomes.
